# Gap formation following climatic events in spatially structured plant communities

**DOI:** 10.1038/srep11721

**Published:** 2015-06-26

**Authors:** Jinbao Liao, Hans J. De Boeck, Zhenqing Li, Ivan Nijs

**Affiliations:** 1 Research Group Plant and Vegetation Ecology, Department of Biology, University of Antwerp (Campus Drie Eiken), Universiteitsplein 1, B-2610 Wilrijk, Belgium; 2 Ministry of Education’s Key Laboratory of Poyang Lake Wetland and Watershed Research, Jiangxi Normal University, Ziyang Road 99, 330022 Nanchang, China; 3 State Key Laboratory of Vegetation and Environmental Change, Institute of Botany, Chinese Academy of Sciences, Beijing 100093, China

## Abstract

Gaps play a crucial role in maintaining species diversity, yet how community structure and composition influence gap formation is still poorly understood. We apply a spatially structured community model to predict how species diversity and intraspecific aggregation shape gap patterns emerging after climatic events, based on species-specific mortality responses. In multispecies communities, average gap size and gap-size diversity increased rapidly with increasing mean mortality once a mortality threshold was exceeded, greatly promoting gap recolonization opportunity. This result was observed at all levels of species richness. Increasing interspecific difference likewise enhanced these metrics, which may promote not only diversity maintenance but also community invasibility, since more diverse niches for both local and exotic species are provided. The richness effects on gap size and gap-size diversity were positive, but only expressed when species were sufficiently different. Surprisingly, while intraspecific clumping strongly promoted gap-size diversity, it hardly influenced average gap size. Species evenness generally reduced gap metrics induced by climatic events, so the typical assumption of maximum evenness in many experiments and models may underestimate community diversity and invasibility. Overall, understanding the factors driving gap formation in spatially structured assemblages can help predict community secondary succession after climatic events.

Gap creation is a common disturbance in many ecosystems^1^,^2^,^3^,^4^,^5^. It maintains species diversity and regulates community succession and dynamics by locally producing a heterogeneous and resource-rich environment in which regeneration is favoured^1^,^4^,^5^,^6^,^7^. Gap-creating processes typically give rise to a wide range of regeneration niches which are suitable for species with different life-histories^1^,^6^,^8^. In addition, the higher levels of light, water and soil nutrients in gaps can promote seedling establishment and sapling densities for local as well as exotic species^4^,^5^,^9^,^10^,^11^.

Which species - either present in the seed bank or imported through exotic invasion - can establish in gaps is to a large extent determined by gap properties. Large gaps and gap centres are often colonized by light-demanding pioneer species^12^,^13^, while small gaps are occupied more by shade-tolerant ones^14^. Small openings are also more frequently filled by clonal growth, whereas successful establishment via seed germination prevails in larger gaps^15^. The recolonization potential is further influenced by gap shape^16^,^17^,^18^, as individuals at gap borders are more exposed to competition with surrounding plants. A longer gap perimeter (i.e., border length) thus tends to compromise survival^19^,^20^.

Because of the ecological significance of gap formation, understanding how individual gap properties and spatial patterns of gaps are generated is a prerequisite to better predicting community succession. Much of gap creation in plant communities is caused by climatic events, such as windstorms, flooding, frost, local fire following lightning, droughts and heat waves. Relationships between the gap patterns arising from such events and biophysical factors (e.g., species composition, age, size, topography, soil, etc.) have been explored in a variety of ecosystems^21^,^22^,^23^,^24^,^25^,^26^,^27^,^28^. Several observations suggest that especially species diversity may play a critical role in shaping the gaps resulting from such perturbations^21^,^22^,^23^,^24^. According to Nijs and Roy^29^ and Mason *et al.*^30^, species diversity can be partitioned into three principal components: species richness, species difference and species evenness. Van Peer *et al.*^21^,^22^ experimentally found that species richness increases mean gap size but reduces gap density in grassland ecosystems exposed to combined drought and heat extremes, due to higher water consumption in species-rich systems. The second component of diversity, species differences in functional traits, may directly affect gap-creating processes when species have divergent tolerances/sensitivities to climatic events^23^,^24^,^31^,^32^,^33^,^34^. For example, many empirical studies reported that the species mortality pattern in forest ecosystems is at least partly due to interspecific variation in susceptibility to windthrow^31^,^32^,^33^,^34^. Less attention has been paid to the role of species evenness in gap formation. As plant communities in nature often exhibit low levels of evenness^35^, common species may dominate the gap creation process. Besides these elements of species diversity, the spatial distribution of the species in the community prior to a climatic event may determine the emerging gap pattern when mortality responses are species-specific.

Despite the likely importance of species diversity and spatial aggregation for the gap patterns that emerge after perturbation, experimental approaches on the subject remain scarce and hardly any models have investigated the mechanisms involved, with the model of Li *et al.*^36^ as a notable exception. However, the synthesized community patterns in this modelling study were too regular to realistically capture the irregularities of natural communities, as intraspecific aggregation was varied by changing the cell size of a chessboard. The orderly mosaic of species clumps in such an ideal matrix approach is unlikely to represent the gap merging processes taking place in real communities upon exposure to perturbation. Furthermore, the effect of species evenness on gap formation was not explored by Li *et al.*^36^, in spite of its significance for ecosystem stability and vulnerability to invasion^37^,^38^,^39^.

Here, we revisit the work of Li *et al.*^36^ by synthesizing multispecies communities with the SIMMAP software^40^,^41^, which simulates more realistic stands compared to the previously applied matrix and chessboard approach. The multispecies communities are subjected to a climatic perturbation that creates gaps in a single step. We examine how species diversity (i.e., richness, difference and evenness) and spatial aggregation (from random to clumping) influence the resulting gap pattern and therefore recolonization opportunity.

## Results

**Figure 1:**
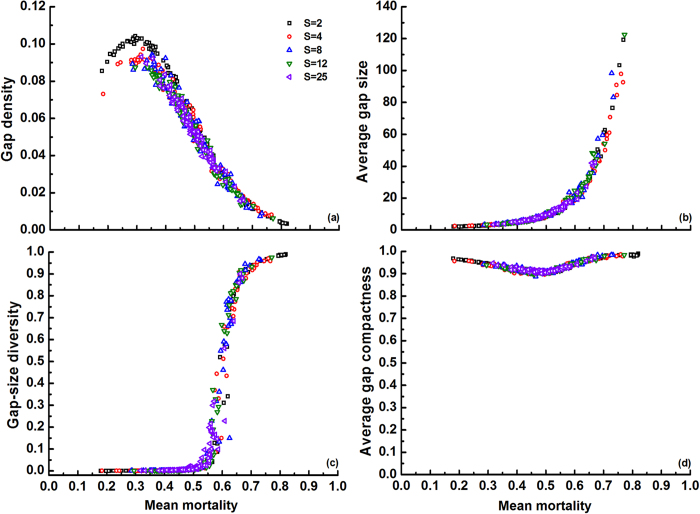
Effects of average species mortality (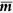) and species richness (*S* = 2, 4, 8, 12 or 25) on four gap metrics (see Table 1) in communities with intermediate degree of intraspecific aggregation (*p* = 0.3). Replicates (100) at each *S* level have randomly produced species mortality rates *m*_*i*_ ∈ [0,1] at the same dispersion of *m*_*i*_ values 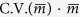 = 0.25 (note that the C.V.(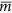) values among communities are different due to varied 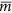, and the range of 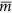 shrinks with increasing *S*). Species evenness *E* was set to 1.

We first investigated how different average mortality rates (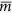) affect gap pattern in plant communities with varying species richness (*S*) but the same dispersion of *m*_*i*_ values 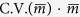 = 0.25 (Fig. 1). Regardless of species richness, gap density first increased with 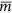 and then decreased because of gap mergence after reaching a peak value at 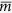 ≈ 0.3 (Fig. 1a). Both average gap size and gap-size diversity were enhanced by 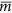, especially above 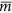 = 0.5 (Fig. 1b,c). Probably, very large gaps are only formed above this threshold, thus maximizing the variety of sizes. Average gap shape compactness, on the other hand, was always high, approximately between 0.9 and 1, with a local minimum at around the same threshold of 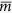 = 0.5 (Fig. 1d). High compactness can be explained by the fact that small gaps, which have the highest compactness, always outnumber the large gaps (this phenomenon is also observed in subsequent simulations). Species richness had almost no influence on gap metrics, which can be understood from the constant 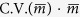 (=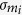) in these simulations. The latter generates a roughly symmetric distribution of the *m*_*i*_ values around the average 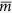 at all richness levels, thus yielding similar gap patterns.

**Figure 2:**
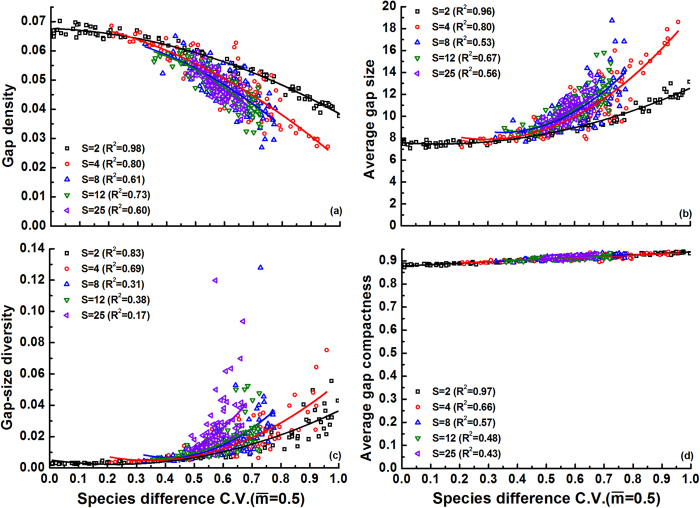
Effects of species variation in mortality (C.V.(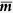)) and species richness (*S* = 2, 4, 8, 12 or 25) on gap metrics in communities with moderate intraspecific clumping (*p* = 0.3). Replicates (100) at each *S* level have randomly generated species mortality rates *m*_*i*_ ∈ [0,1] around the mean 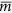 = 0.5 (note that C.V.(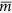) shrinks with increasing *S*). Polynomial fitted curves were significant at each richness level (P < 0.01). Species evenness *E* was set to 1.

Secondly, we examined the effects of species difference (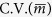 with 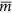 = 0.5) and species richness (*S*) on gap formation in communities with medium intraspecific clumping *p* = 0.3 (Fig. 2). Increasing mortality differences enhanced gap size, gap-size diversity and gap shape compactness, while gap density declined, regardless of species richness. Clearly, increasing C.V.(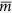) by expanding the range of *m*_*i*_ values adds species with both lower and higher *m*_*i*_ (relative to 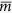 = 0.5) to the community. Species with lower *m*_*i*_ (<0.5) always create small gaps, while species with higher *m*_*i*_ (>0.5) promote gap mergence within and between species clumps (see Fig. 1), ultimately leading to greater gap size and gap-size diversity (Fig. 2b,c). Correspondingly, gap density was reduced since the total gap area (=gap density × gap size) was kept constant at 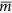 = 0.5 throughout the entire interspecific variation range tested (Fig. 2a). Species richness increased gap size and gap-size diversity, while it reduced gap density. These effects were expressed especially under large interspecific variation in mortality (C.V.(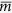) > 0.5), but the richness effects on gap size and density saturated at higher *S*. Logically, increasing richness at the same C.V.(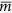) generates a wider range of *m*_*i*_ values, thus increasing gap size and lowering gap density. The gradual weakening of these richness effects (Fig. 2a,b) probably arises from the saturating range of *m*_*i*_ values at the fixed C.V.(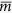) used. Similar to Fig. 1, species richness hardly influenced gap compactness (Fig. 2d). Considering both species richness and difference, we conclude that communities with more species that vary widely in mortality, are generally left with fewer but on average larger and more diverse gaps after a climatic event, while gap shape compactness is little influenced.

**Figure 3:**
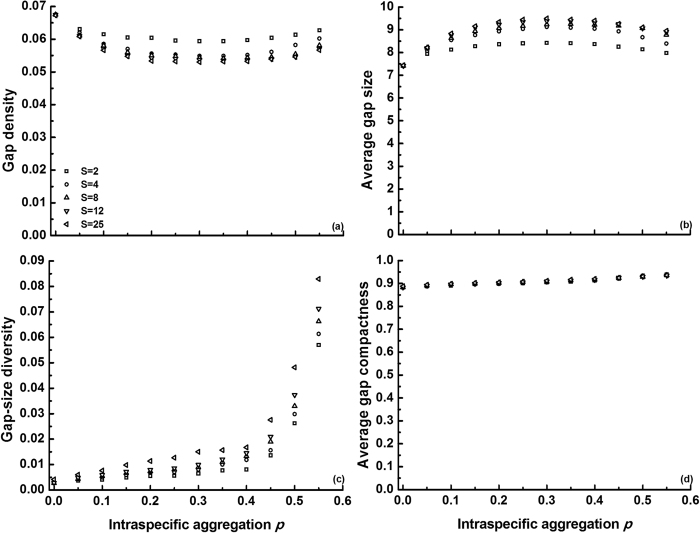
Effects of intraspecific clumping (*p*) and species richness (*S* = 2, 4, 8, 12 or 25) on gap metrics (average of 100 replicates) again at maximum *E* = 1. In each replicate, species mortality rates *m*_*i*_ were randomly produced at 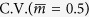 = 0.5. Standard deviations (SDs) were omitted for clarity (note that SD increases with *p*): (a) 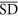 = 0.00347; (b) 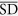 = 0.60234; (c) 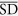 = 0.00756 and (d) 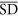 = 0.00445.

Thirdly, we investigated how intraspecific clumping (*p*) modulates gap pattern (Fig. 3, simulations at medium species difference). Regardless of species richness, the gap density – *p* relationship exhibited a minimum at *p* = 0.3, whereas gap size peaked at this clumping degree (Fig. 3a,b). While these effects were weak, intraspecific aggregation promoted gap-size diversity, especially above *p* = 0.4 (Fig. 3c). Obviously, increasing *p* enhances gap mergence within conspecific clumps, especially for species with high *m*_*i*_ (>0.5) values (see Fig. 1). Again, gap shape compactness was high throughout and fairly insensitive (to *p*) (Fig. 3d). Species richness affected gap metrics as in Fig. 2. Richness effects were absent however at the lowest clumping degree *p* = 0 (i.e., in a random community), as a random procedure then determines whether individuals die or not, thus leading to the random gap pattern.

**Figure 4:**
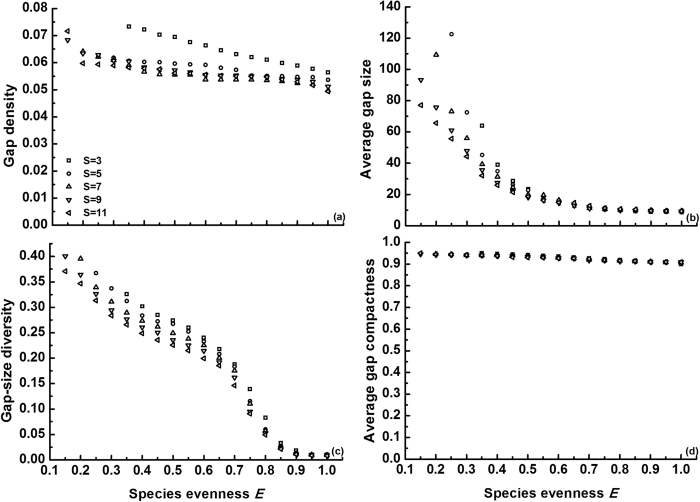
Effects of species evenness (*E*) and species richness (*S* = 3, 5, 7, 9 or 11) on gap metrics (mean of 100 replicates) in communities with medium intraspecific aggregation (*p* = 0.3). Species unevenness was created by increasing the population size for a dominant species, while the remaining individuals were equiproportionally allocated to the subdominant species (see Methods). Note that the range of *E* expands with increasing *S* (see equation (1)). Similar to Fig. 3, we randomly generated mortality rates under 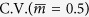 = 0.5 in each replicate, in order to eliminate the effect of the dominant species identity (i.e., mortality trait) on gap formation. Curves for different *S* levels cannot be compared directly, as the clump size of the dominant is different, with lower richness causing larger clump size. Error bars (SDs) were omitted for clarity (note that SD decreases with *E*): (a) 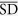 = 0.02735; (b) 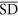 = 39.24072; (c) 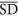 = 0.26998 and (d) 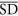 = 0.02315.

**Figure 5:**
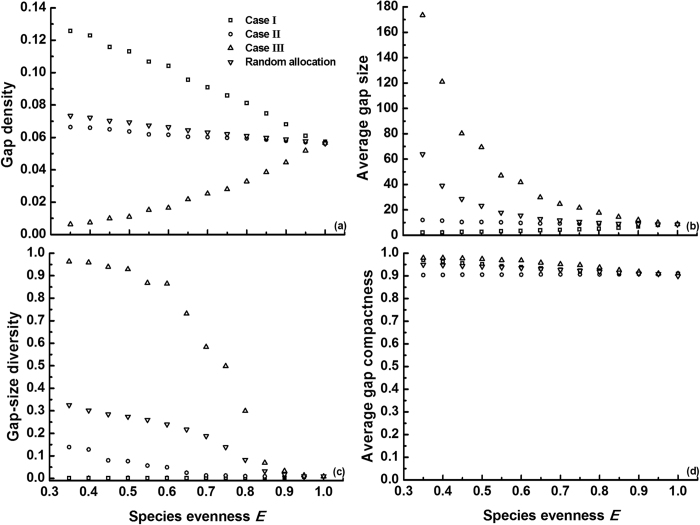
Effect of the identity of the dominant species (i.e., mortality trait) on gap metrics (mean of 100 replicates) at varying evenness *E* and species richness *S* = 3. For comparison, the gap metrics obtained from the random mortality assignment in Fig. 4 are also included. Likewise to Fig. 4, mortality values were randomly generated at 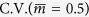 = 0.5, but three types of species mortality allocation were simulated: (I) dominant species with lowest *m*_*i*_, (II) dominant species with median *m*_*i*_, and (III) dominant species with highest *m*_*i*_ (see Methods). Intraspecific aggregation was set to *p* *=* 0.3. SDs of replicates are omitted for clarity (SD decreases with *E*, and higher *m*_*i*_ of the dominant causes larger SD): (a) 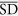 = 0.00785; (b) 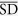 = 7.15210; (c) 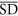 = 0.06231 and (d) 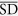 = 0.00888.

Next, we tested how species evenness (*E*) determines gap formation induced by climatic events. These simulations were done at intermediate values of the other parameters, again including variation in species richness (Fig. 4). In general, the impact of species evenness (100 replicates with random mortality assignment) was negative for all gap metrics, especially for gap size and gap-size diversity. To explain this we focus on the low evenness levels, where the identity (i.e., mortality trait) of the most abundant species undoubtedly dominates gap formation. If, by chance, a high mortality is allocated to this dominant species, large gaps are created and therefore also a high gap-size diversity. Dominant species with a low mortality, on the other hand, can only create small gaps and thus low gap-size diversity (see also Fig. 5). As species evenness increases, the impact of the dominant species obviously weakens, hence the declining metrics. Unlike the aforementioned cases, species richness generally reduced gap metrics. This can be understood from the fact that a different *S* at the same *E* changes the clump size of the dominant (due to different relative abundances), with lower richness resulting in greater clump size of this dominant (see Methods) and thus larger gaps after mortality. These richness effects gradually disappear towards *E* = 1, because differences in clump size progressively fade.

Finally, we explored how the identity (mortality trait) of the dominant species modulates gap pattern with increasing *E* (Fig. 5), in order to analyze more in depth how the evenness effects in Fig. 4 are brought about. In case I (dominant species with the lowest mortality *m*_*i*_), increasing *E* linearly reduced gap density, while gap size and gap-size diversity were very low and hardly responsive to *E*. Obviously, a large number of small gaps are generated by allocating the lowest *m*_*i*_ to the dominant species in a very uneven community, and these gaps will become less abundant with *E* through a declining relative abundance of this dominant. When assigning the median *m*_*i*_ to the dominant (case II), species evenness weakly decreased gap density and gap-size diversity, whereas gap size was almost not influenced. In contrast to case I, species evenness in case III (dominant species with highest *m*_*i*_) substantially reduced gap size and gap-size diversity but increased gap density. Note that the gap metrics in case II (dominant with median *m*_*i*_) deviated from those obtained with random mortality assignment (average of 100 replicates), especially for gap size and gap-size diversity. Different mortality traits for the dominant thus contributed disproportionately to the averaged evenness effect on gap formation (Fig. 5).

## Discussion

Similar to Li *et al.*^36^, we simulated gap formation in multispecies communities from a “static” perspective, by assigning species-specific mortalities that lead to gap creation in a single step. These communities were assumed to be disturbed by climatic events that occur within a short time frame and have rapid and major impact on plant survival. We only considered ‘global’ disturbance by defining that all individuals in the entire landscape are disturbed, while ignoring possible spatial variability in disturbance (e.g., localized disturbance). This is because many climatic events exhibit a global character, such as frost, flooding, drought and heatwaves. By using more realistic communities characterized by patches of irregular shape as in natural stands, we obtained both similar and different results compared with Li *et al.*^36^.

According to Li *et al.*^36^, species richness and interspecific differences promote gap size, while gap-size diversity is enhanced both by interspecific differences and by intraspecific clumping. Our model confirms these previous findings, based on the same mechanisms. Another similarity between both studies is the high gap shape compactness that we observed regardless of community characteristics (Figs 1, 2, 3, 4, 5). In this case the causes are different though: in Li *et al.*^36^ compact gaps were generated by the regular community matrices that were used, while our result of high gap compactness most likely resulted from the dominance of small gaps (with high compactness). The current model also indicates that small changes in the mean mortality of the species in a multispecies community can disproportionately modify gap size and gap-size diversity, provided a given threshold is crossed (Fig. 1). These percolation effects suggest that subtle variations in species sensitivity to perturbation, or in perturbation intensity, can greatly determine gap recolonization. Li *et al.*^36^ also observed this, but modelled it only for monocultures. Our finding that species-rich systems exhibit the same response and approximately the same threshold, implies that diversity losses should have no major effects on this phenomenon (unless they alter species aggregation or interspecific difference, which were kept constant in these simulations).

Going beyond previous results, we found positive effects of species richness on gap-size diversity, and saturating richness effects on gap size at higher richness levels (Figs 2 and 3, see also further). These results were opposite in Li *et al.*^36^, due to an unjustified setting of C.V.(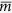) = 1 in the corresponding analyses (i.e., assigning *m*_*i*_ = 0 to half of the species and *m*_*i*_ = 1 to the other half). In fact, setting C.V.(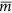) = 1 effectively reduces species richness so that only two species were considered in their simulation, one with *m*_*i*_ = 0 and another with *m*_*i*_ = 1. Testing for *S* effects under these conditions thus becomes meaningless. By setting C.V.(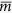) = 1, Li *et al.*^36^ also erroneously concluded that intraspecific clumping promotes gap size. Here we show that, under medium interspecific difference, increasing conspecific clumping tends to shape large numbers of small gaps in species with low mortality traits (*m*_*i*_ < 0.5), but at the same time large gaps in species with high mortality rates (*m*_*i*_ > 0.5) owing to gap mergence within clumps (see Fig. 1). As a result, average gap size becomes fairly independent of clumping degree (Fig. 3). Extrapolation from Li *et al.*^36^ may thus result in erroneous estimation of gap recolonization opportunity in some conditions (see also further).

Both species richness and difference enhanced gap size and gap-size diversity, as confirmed in the experimental study of Van Peer *et al.*^21^ for richness effects on average gap size. However, these positive richness effects were only expressed when species were sufficiently different (C.V.(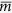) > 0.5, Fig. 2). Larger and more diverse gaps may provide greater opportunities for colonizers with different niches, thereby promoting diversity maintenance^6^,^42^,^43^. For example, species with divergent functional traits (shade tolerance, competitive abilities, reproductive traits, etc.) often differ in their ability to exploit gaps of various sizes^14^,^15^,^44^,^45^,^46^. However, it should be emphasized that effects of increasing species richness on gap size became progressively smaller (Figs 2 and 3), suggesting that very high species richness levels such as, for example, in tropical forests may not influence local colonization opportunity and invasion possibility for exotic species any further. Interestingly, these saturating richness effects on gap metrics are reminiscent of biodiversity-ecosystem functioning relationships which likewise typically plateau at high richness levels owing to functional redundancy^47^,^48^.

Species richness and difference promoting gap size and gap-size diversity might also make plant communities more susceptible to invasion, indirectly supporting the positive correlation between richness and invasibility^49^,^50^. Large gaps may result in large amounts of unused resources, providing a resource-rich niche that alien species can colonize^51^. In addition, alien species in larger gaps would undergo reduced competition from local species. In contrast, many studies have found that species richness may reduce invasibility due to resource-use complementarity^52^,^53^,^54^,^55^, i.e., more complete resource use resulting in fewer resources being available for invaders^53^. The different richness-invasibility relationships are probably a spatial scale issue^52^: species-richer assemblages might be at greater risk of invasion at large scales (e.g., landscape) because the same large-scale drivers promote native and alien richness alike, while species richness at small scales (e.g., neighbourhood-scale) may enhance invasion resistance due to the dominance of the spatial complementarity mechanism. While the large-scale patterns may include the positive effect of species richness on gap size and gap-size diversity reported here because the influence of climatic events would be included in these observations, experiments focusing on the small scale may have missed this novel link between species richness and invasibility because climatic events causing gap formation are rarely part of such experiments.

While average gap size was fairly independent of clumping degree, intraspecific clumping strongly promoted gap-size diversity (Fig. 3). Aggregated communities may thus provide more chances for a variety of colonizers filling the gaps emerging after perturbation (including local and exotic species). Interestingly, gap size and gap-size diversity were not influenced by species richness in random communities (*p* = 0 in Fig. 3b,c), implying that losing or adding species in those systems would not change the opportunities for colonizers. Probably, randomly mixed species also lead to fully randomized gap patterns across the simulated community^21^,^36^. Furthermore, the randomly synthesized communities had minimal gap-size diversity relative to more realistic communities with some degree of intraspecific aggregation. This suggests that studies on gap dynamics in random communities might underestimate community diversity and invasibility.

Species evenness exerted a negative effect on all gap metrics (Fig. 4), implying that gap recolonization after climatic events would be hampered in equitable communities because of less available locations and a reduced range of potential colonizers. Indirectly, this finding also lends support to the concept of negative evenness-invasibility relationships^39^,^56^,^57^. According to these empirical studies, a community with high evenness, similar to a community with high richness, is reputed to utilize resources more fully through complementarity, which reduces invasibility. In addition, the probability that a species resistant to invaders is present, is greater in evenly composed communities (selection effect), further enhancing community resistance to invasion. Here we propose an alternative mechanism to explain the negative relationship: in the smaller gaps associated with high species evenness, invaders would face stronger resource competition with local species. Despite its importance to community stability, species evenness has been ignored in most previous studies which have typically set *E* = 1 by default. This may have led to underestimation of invasibility in the real world, as natural communities are always characterized by some degree of species unevenness^58^.

In a single community, the mortality of the dominant species determined the evenness effect (positive or negative) on gap metrics (Fig. 5). Dominant species that are sensitive to the perturbation (case III) may promote both species coexistence and community invasibility by increasing gap size and diversity. A low-sensitive dominant (case I), on the other hand, would create small gaps which may reduce species coexistence but enhance community resistance to invasion. This may explain why some - natural or experimental - uneven communities exhibit greater susceptibility to invasion while others show more resistance^57^,^59^. Interestingly, attributing a median mortality to the dominant (case II) generated a different gap pattern compared to random mortality assignment (Fig. 5). The effect of a dominant species with mean traits thus cannot be used to represent the averaged evenness impact on gap formation. Therefore, dominant species identity, rather than community evenness, may be a key to explaining invasibility in real communities (which normally deviate substantially from *E* = 1)^55^,^60^,^61^.

In summary, based on species-specific mortality responses, we modelled the effects of species diversity (i.e., richness, evenness and difference) and intraspecific aggregation on gap formation in spatially structured assemblages characterized by patches of irregular shape. These community characteristics have the potential to significantly alter the gap patterns and consequently the recolonization opportunities emerging after climatic events. Our simulations particularly demonstrated that the species evenness of the community and the sensitivity of the dominant species are critical for diversity maintenance and community invasibility. Future work could extend our ‘static’ approach where gap formation was simulated in a single step, to modelling community dynamics in response to climatic events. Further study could also focus on model validation, which can be achieved by different means. Van Peer *et al.*^21^,^22^ already studied gap formation resulting from experimentally imposed extreme drought and heat in synthesized grassland mesocosms, though only to explore the effect of species richness on the emerging spatial mortality pattern. Extending this type of experiment to test the role of other initial community characteristics, for example, of similarity in species traits (by planting combinations of highly similar or very different species) or of intraspecific aggregation (by manipulating the initial spatial distribution of the plants) would be fairly straightforward. Another approach would be to collect data on species mortality pattern in plant communities in the field following a climatic event, recording species identity, mortality and spatial distribution in quadrats and estimating the interspecific differences in tolerance to the perturbation from the data analysis.

## Methods

### Community structure

We simulate multispecies communities with a two-dimensional square lattice of size *L* *×* *L* = 200 × 200 cells, where *L* is the length of the lattice. Each cell is occupied by an individual belonging to one of the species. Using the landscape simulation software SIMMAP based on a modified random clusters method^40^,^41^, we vary three parameters as follows:

1. Species richness (*S*) is varied by directly setting the species number in SIMMAP. When all species have the same population size, each species has *L*^2^/*S* individuals.
2. Species evenness (*E*), with 0 < *E* ≤ 1, describes the equitability of the species’ relative abundances^62^, which can be generated by adjusting each species relative abundance. There are many metrics of species evenness, each of which has its pros and cons^61^,^63^,^64^. Here we use the Gini-index *G* ∈ [0,1]^65^, which proved to be a useful quantification of population size variability^66^. Following Weiner and Solbrig^67^,
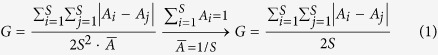
where *A*_*i*_ (or *A*_*j*_) is the relative abundance of species *i* (or *j*), and 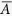 denotes the average relative abundance of all species, with 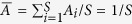. The virtue of *G* is that it is based on the Lorenz curve which describes distribution equity as the relationship between the cumulative proportion of species richness and the cumulative proportion of species abundance, with a higher *G* corresponding to a more uneven community^66^,^68^. Therefore, species evenness can be quantified as *E* = 1-*G*.
3. Intraspecific aggregation (*p*), with 0 ≤ *p* ≤ *p*_*c*_, expresses the intraspecific clustering degree, where *p* = 0 denotes a randomly structured community with minimal intraspecific aggregation, and the percolation threshold *p*_*c *_≈ 0.5928 represents the upper-bound of intraspecific clumping under the default 4-neighbour principle^41^.

**Figure 6:**
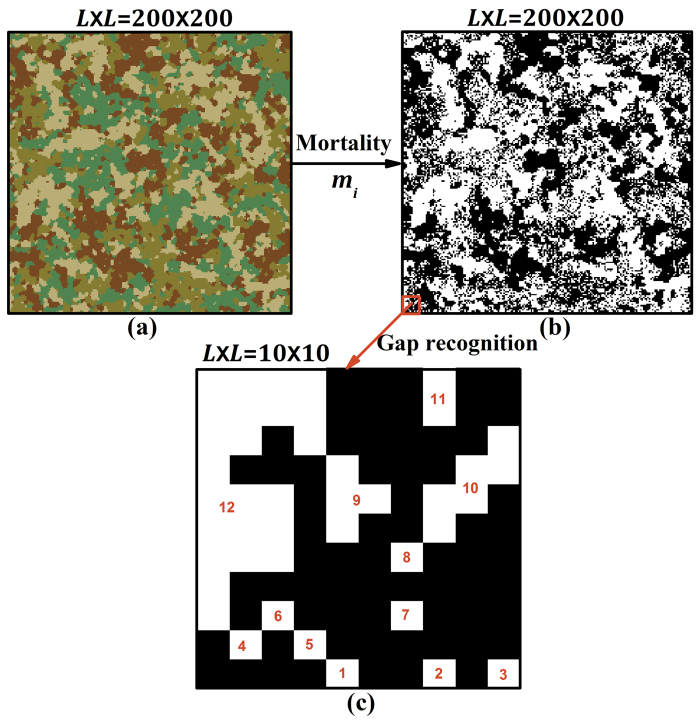
Illustration of gap formation and recognition. (**a**) Community of 200 × 200 cells with four species (colours); (**b**) Gap formation after assigning species-specific mortality (white – empty cells, and black – occupied cells); (**c**) Gap recognition marked with numbers, following the principle of four nearest neighbours. Parameters: species richness *S* = 4, species evenness *E* = 1, intraspecific clumping *p* = 0.5, and species mortality rates *m*_*i*_ ∈ {0, 0.3, 0.7, 1} with mean mortality 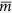 = 0.5.

By fixing the above parameters and otherwise allocating the species randomly to the cells of the matrix to represent natural stochasticity, the SIMMAP software can assemble the patchy and irregular communities that are typical for many landscapes (see Fig. 6), without defining specific landscape processes^41^. Furthermore, the software allows the simulation of a wide range of spatial community patterns, with independent variation of community characteristics. For example, communities can be simulated with all possible degrees of intraspecific clumping (ranging from random to high degrees of intraspecific aggregation) and species evenness (between 0 and 1). The simulations are also generic, representing vegetation in general provided plants largely cover the soil (e.g., temperate and tropical forest, temperate grassland, savanna, etc.). For these reasons, SIMMAP has been widely applied to simulate and analyze plant communities^69^,^70^,^71^,^72^. Given the broad array of simulated communities in the current study, resulting from the systematic variation of their properties, some communities will be common in nature and others will be less common. Establishing the latter was not our goal, instead we provide a catalogue of community patterns from which ecologists who study a particular system and would like to analyze how climatic events might alter it, can select the most relevant case.

### Gap formation

We simulate a severe perturbation (climatic event) in multispecies communities by applying species-specific mortality probabilities *m*_*i*_ ∈ [0,1] in a single step (Fig. 6), and explore the resulting gap pattern from a static perspective (i.e., we do not model long-term gap dynamics under disturbance regimes). Within a single step, each individual’s mortality is determined by comparing its *m*_*i*_ with a randomly generated *r* ∈ [0,1]. The individual is assumed to die if *r* ≤ *m*_*i*_ and survive otherwise. This makes our study most relevant for communities subjected to climatic events that kill part of the plants on a relatively short timescale (i.e., pulse disturbance). In our model, mortality is varied only between species, ignoring intraspecific variation in *m*_*i*_. We include the full range of species mortality between 0 and 1 to reflect that some species can be very sensitive in the face of climatic events (most individuals dying), while others are very robust (most individuals surviving), as empirically confirmed by Dreesen *et al.*^73^ for drought and heat extremes. Disturbance intensity being mild or severe likewise justifies using the maximum possible range. Species difference in mortality owing to different sensitivity to perturbation, calculated as the coefficient of variation 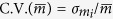 (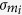
as standard deviation measures the dispersion of a set of *m*_*i*_ values), characterizes the relative dispersion of species mortality rates *m*_*i*_ around the mean mortality 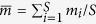. Varying 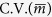 is realized by enlarging or shrinking the range of *m*_*i*_ values around the average 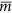. Like many simulations in ecology, our models omit some biological detail so as to preserve generality and transparency. For example, possible neighbouring effects on gap formation such as treefall affecting adjacent trees, are ignored, so in these cases the simulations should be used with caution.

### Gap metrics

Gap patterns are analyzed with the FRAGSTATS software^74^, which recognizes each gap based on the principle of 4-nearest neighbours (see Fig. 6). In other words, an empty cell in a square lattice is treated as an isolated gap only if its four nearest neighbouring cells are occupied by individuals; otherwise it is considered as part of another gap (Fig. 6). After gap recognition, we apply gap metrics to analyze the statistical properties of the whole gap pattern. From the large number of landscape indices that have been formulated to describe spatial gap pattern^75^,^76^,^77^, we select four indices (see details in Table 1): gap density, average gap size, gap-size diversity^77^, and average gap-shape compactness^78^. These metrics, of which the potential ecological significance is listed in Table 1, can adequately characterize the fundamental and independent components of gap pattern.

### Simulation cases

We first investigated how variation in the average mortality of all species (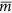) affects gap metrics at different richness levels (*S* = 2, 4, 8, 12 and 25), while keeping species evenness at its maximum (*E* = 1) and intraspecific clumping at a medium level of *p* = 0.3. At each richness level, we generated 100 communities with *p* = 0.3. In each community, the species mortality values *m*_*i*_ ∈ [0,1] were randomly produced under a specified range of species variation 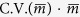 = 0.25 (i.e., fixed dispersion 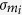 = 0.25), thereby yielding different 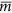 values among communities. Note that the values of C.V.(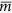) (=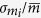) – relative range of *m*_*i*_ values around 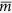 – among communities are different because of varied 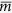.

Secondly, we tested the effect of species difference C.V.(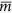) on gap metrics at different richness levels (*S* = 2, 4, 8, 12 and 25). For each richness level, we again produced 100 communities at *p* = 0.3, but each community received a different C.V.(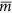) by allocating randomly generated mortality values *m*_*i*_ ∈ [0,1] around the fixed mean 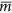 = 0.5 to the species. *E* was set to 1 as in the first case.

In a third set of simulations, we examined how intraspecific aggregation (*p*) affects gap metrics. Species difference was set to a medium C.V.(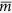 = 0.5) = 0.5 while varying species richness (*S* = 2, 4, 8, 12 and 25) and maintaining *E* = 1. Each case was explored with 100 replicates, starting from different community patterns in each replicate, but with the same distributed properties (i.e., *S*, *E* and *p*). In each replicate, species mortality rates *m*_*i*_ were randomly produced under the condition 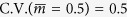. Ultimately, the average of these 100 replicates yielded the mean gap metrics.

Next, we explored the influence of species evenness (*E*) on gap metrics at different species richness levels (*S* = 3, 5, 7, 9 and 11), while keeping *p* = 0.3 and 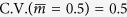. Evenness can be generated in numerous ways; here we simply varied *E* by changing the number of individuals in the most abundant species, while uniformly allocating the remaining individuals to the other *S*-1 species^29^,^79^. When all species have the same population size (*L*^2^/*S*), the maximal *E* = 1 is obtained. In contrast, when *L*^2^-*S* + 1 individuals are allocated to the dominant species, and one individual to each of the remaining (*S*-1) species, the minimal *E* is achieved. The allowable minimum *E* thus decreases with increasing richness. When *E* is varied, it is important to determine which mortality trait is allocated to which species. For example, assigning the highest mortality rate to the dominant species or to one of the subdominant species, results in different gap patterns. To eliminate this selection effect of species identity (i.e., mortality traits) on gap formation, we ran each case with 100 replicates by allocating randomly generated *m*_*i*_ values (under 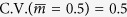) to the species in each replicate, similar to the third set of simulations above. This design has already been used for exploring the evenness effect on ecosystem functioning^79^.

Finally, we examined the impact of the identity (i.e., mortality trait) of the dominant species on gap metrics while varying evenness at *S* = 3 and *p* = 0.3, in order to compare the outcome with the case where the mortality traits were randomly assigned (see above). This explores how different species mortality assignments lead to different gap patterns, and whether any of these assignments can represent the evenness effect on gap formation under random assignment. Based on the randomly generated *m*_*i*_ values at 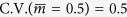, three assignments were simulated: (I) dominant species with lowest *m*_*i*_, (II) dominant species with median *m*_*i*_, and (III) dominant species with highest *m*_*i*_, while the remaining *m*_*i*_ values were always randomly allocated to the subdominant species (with each the same population size). We present the case of *S* = 3 as an example, since it yielded similar outcomes as cases with *S* > 3. Again, each case was simulated with 100 replicates, starting from different community patterns in each replicate.

## Additional Information

**How to cite this article**: Liao, J. *et al.* Gap formation following climatic events in spatially structured plant communities. *Sci. Rep.* **5**, 11721; doi: 10.1038/srep11721 (2015).

## Figures and Tables

**Table 1:** Four metrics of gap patterns.

Indices	Formula	Definition	Ecological significance
Gap density^75^	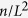	Number of gaps per unit area, with gap numbers divided by lattice size	Reflects sites’ availability for colonization
Average gap size^77^	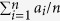	Mean of gap area based on cell counts	Indicates local colonization opportunity and invasion possibility for exotic species
Gap-size diversity^36^,^77^	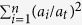	The inverse of Simpson’s index, with high gap-size diversity being expected when few large gaps are present, and/or when the gaps have uneven sizes	Estimates the potential species range of colonizers filling these gaps
Average gap-shape compactness^78^	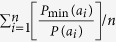	Average of the ratio of the minimum perimeter for each gap to the actual perimeter, reflecting the deviation of the gap’s shape from a perfectly isodiametric one	Describes the interactions of potential colonizers (local and exotic) with the local community

Parameters: *n-*total gap number, *L-*length of the simulated lattice, *a*_*i*_*-*the *i-*th gap size, *a*_*t*_*-*total area of all gaps with 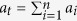, 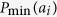 *-*minimum gap perimeter for the *i-*th gap with size *a*_*i*_, 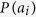
*-*actual gap perimeter of the *i-*th gap.
